# Advancing one health implementation in the Eastern Mediterranean region: insights from the EMPHNET 8th biennial roundtable

**DOI:** 10.3389/fpubh.2025.1652846

**Published:** 2025-11-17

**Authors:** Sayed Himatt, Muna Abdel Aziz, Baher ElDesouki, Marion Muehlen, Mazen Malkawi, Julio Pinto, Mohannad Al Nsour, Yousef Khader, Haitham Bashier, Shukri Adam, Ibrahim Al Faouri

**Affiliations:** 1 The Eastern Mediterranean Public Health Network (EMPHNET), Amman, Jordan; 2 Public Health for Salford, Salford, United Kingdom; 3 YourB Credit Union Ltd., Manchester, United Kingdom; 4 One Health Expert, Preventive Medicine and Public Health Sector, Ministry of Health and Population, Cairo, Egypt; 5 Surveillance Systems/Field Epidemiology Capacity, Strengthening, World Health Organization (WHO), Geneva, Switzerland; 6 Public Health Department, Ministry of Health and Prevention, Dubai, United Arab Emirates; 7 Animal Production and Health Division, Food and Agriculture Organization of the United Nations (FAO), Liaison Office with the United Nations, Geneva, Switzerland; 8 Department of Public Health, Jordan University of Science and Technology, Irbid, Jordan; 9 RAK Medical & Health Sciences University, Ras Al-Khaimah, United Arab Emirates

**Keywords:** one health appraoch, Eastern Mediterranean region (EMR), workforce development, surveillance, multisectoral collaboration

## Abstract

Countries in the Eastern Mediterranean region (EMR) face interconnected risks at the human, animal, and environmental interface. This Perspective synthesizes lessons from a roundtable convened during the EMPHNET 8th Biennial Regional Conference (Amman, 15–18 September 2024) to identify near-term actions to operationalize One Health. The two-hour session featured five presentations, a panel, and open discussion with 68 participants from human, animal, and environmental health, food safety, life sciences, and social sciences. Rapporteur notes and slides were independently reviewed by two authors and consolidated into cross-cutting themes. Five priorities emerged: workforce development and mentorship; governance and multisectoral coordination; surveillance, data integration, and joint risk assessment; financing and sustainability; and climate and environmental determinants. Participants emphasized Competencies for One Health Field Epidemiology (COHFE)-aligned competency pathways, institutionalized coordination with defined roles, interoperable surveillance products using shared case definitions, early joint risk assessments with explicit triggers, and sustained cross-sector rapid response capacity. They called for embedding One Health tasks in national budgets, aligning external support to government plans, and integrating health within climate policies and investments. Egypt case illustrated national organization of governance, surveillance, and financing. The roundtable offers a practice-oriented entry point for EMR decision makers to operationalize One Health in the near term.

## Introduction

The One Health approach, as defined by the One Health High-Level Expert Panel (OHHLEP), optimizes human, animal, and ecosystem health by addressing their interdependence; it integrates human–animal–environmental domains across sectors and levels and supports safe food, water, energy, and air quality in pursuit of the Sustainable Development Goals (SDGs) (1, 2).

COVID-19 exposed systemic vulnerabilities and the need for integrated surveillance and coordinated responses; shifting from a human-only focus to a One Health approach is essential to address interconnected threats (3, 4). One Health initiatives strengthen systems by integrating surveillance and control of zoonoses, Neglected Tropical Diseases (NTDs), vector-borne diseases, food safety, and antimicrobial resistance, while embedding environmental considerations in public health planning (4–7).

The Eastern Mediterranean region (EMR) faces protracted emergencies, displacement, close human–animal contact, and climate stress. Coordination mechanisms exist but perform unevenly. Countries need interoperable surveillance, routine joint risk assessment with agreed triggers, and a standing cross-sector rapid response capacity (4, 6–9).

The Eastern Mediterranean Public Health Network (EMPHNET) advances One Health through its One Health Unit and partners. At the 8th Biennial Regional Conference, EMPHNET convened Roundtable 3, “One Health Approach in the EMR: Challenges, Initiatives, and the Way Forward” (16 September 2024, Amman), to translate recent experience into coordinated action. The session gathered national authorities, Quadripartite partners, academia, and practitioners, strengthening collaboration and resilience.

The roundtable reviewed initiatives and tools, identified shared implementation challenges, and agreed near-term steps: routine joint risk assessment, interoperable surveillance, and sustained cross-sector rapid response capacity.

This Perspective distills session insights for accelerating One Health in the EMR. Egypt’s experience illustrates alignment of governance, surveillance, and financing nationally. It offers a concise, practice-oriented entry point for EMR policymakers and program leads.

## Methods

This Perspective synthesizes a single roundtable held during the EMPHNET 8th Biennial Regional Conference (Amman, Jordan; September 15–18, 2024). The two-hour session included five presentations, a moderated panel, and open Q&A. Approximately 68 attendees were observed, spanning human, animal, and environmental health, as well as food safety, life sciences, and social sciences. As the session was an open parallel event without room-level sign-in, audience composition cannot be formally enumerated.

Two rapporteurs used a structured template to capture agenda topics, examples, and implementation enablers and barriers. After the conference, two authors independently reviewed notes and slides and reconciled a consolidated summary. No inter-rater statistics or participant validation were performed. To organize content, all topics raised across the talks, panel, and Q&A were listed, grouped, and distilled into five cross-cutting themes: workforce development and mentorship; governance and coordination; surveillance, data integration, and joint risk assessment; financing and sustainability; and climate and environmental determinants. Points raised only once were retained as illustrative examples.

Egypt’s experience is presented in Box 1 as a comprehensive multisector example aligned with several themes. Insights are reported in aggregate without attribution, and no identifiable personal information was collected.

## Findings

To support traceability from agenda to synthesis, we note how session components informed the themes. CDC’s FETP-Frontline 3.0 chiefly informed workforce (with links to surveillance). WHO’s COHFE framework informed workforce and governance. FAO’s veterinary/lab capacity work informed workforce, surveillance/data integration, and financing. WHO’s climate segment informed the climate/environment theme with governance links. The Egypt case informed governance, surveillance/JRA, and financing. Panel/Q&A emphasized data sharing, explicit JRA triggers, and sustained cross-sector rapid response.

### Workforce development and mentorship models

This theme synthesizes roundtable content on The Competencies for One Health Field Epidemiology (COHFE) Framework, cross-sector training pipelines [Field Epidemiology Training Program (FETP), Field Epidemiology Training Programs for Veterinarians (FETPV) / In-Service Applied Veterinary Epidemiology Training (ISAVET)], and mentorship models. The One Health approach, aligned with the SDGs, addresses challenges like poverty, climate change, and health inequities through collaboration across public health, veterinary medicine, environmental sciences, and related fields (10–13). Workforce development is hindered by siloed policies, professional segregation, and limited inclusion of environmental and social sciences; curricula often overlook WASH and environmental degradation, weakening the response to drivers such as deforestation and unsustainable agriculture (10, 12, 14).

The One Health Joint Plan of Action (2022–2026) emphasizes competency-based training, mentorship, and data-sharing tools to enhance interdisciplinary collaboration. Core competencies include leadership, communication, systems thinking, and technical skills in outbreak management and disease surveillance. To build a sustainable workforce, it is essential to address gaps in teamwork, improve gender balance, recruit younger professionals, and provide career incentives (12, 14–16).

The FETP, initiated by the U.S. Centers for Disease Control and Prevention (CDC) in 1951 and modeled on the Epidemic Intelligence Service (EIS), has trained over 22,000 public health professionals worldwide (13–15). By December 2023, 98 FETPs operated in more than 200 countries and territories, equipping participants with skills in field epidemiology, outbreak response, surveillance, and evidence-based policymaking (17). Since 2009, veterinarians have been included in FETPs to address human-animal-environment health challenges through One Health principles (18).

The FETP-Frontline 3.0 iteration broadens participation (e.g., laboratorians, para-veterinarians, animal husbandry and WASH personnel) and emphasizes cross-sector collaboration to strengthen outbreak management (19). A 2023 multi-country pilot reinforced mentorship and collaborative practice; persistent challenges include funding dependency, political instability, and limited inclusion of veterinary and environmental sectors, highlighting the need for sustained national support and broader recruitment (20).

Veterinary epidemiology capacity is being advanced through specialized programs. Countries have expanded FETPs to include veterinarians or launched FETPVs in several regions (21, 22).

In 2018, the FAO developed 13 core competencies for field veterinary epidemiology provide a common scaffold, while ISAVET has trained over 1,000 veterinary paraprofessionals and uses tools such as TOM and VLCs to extend reach; future priorities include integrating IT, disease intelligence, and AMR surveillance within nationally led, sustainable implementation (22).

The COHFE Framework, launched in 2024 by FAO, WHO, and WOAH, provides an internationally aligned approach to workforce capacity at the human–animal–environment interface, linking to the Quadripartite Joint Plan of Action, IHR (2005), and the PVS Pathway; it outlines 14 technical and functional domains across training levels, including surveillance, outbreak response, laboratory capacity, leadership, communication, and ecosystem health, and is designed to complement FETPs, FETPVs, FELTPs, ISAVET, FTP-WEBE, and Nature for Health. COHFE supports harmonized curricula, mentorship, certification, and continuing education, aiming to strengthen surveillance and enable coordinated responses to complex threats, thereby contributing to resilient, One Health–oriented systems (23).

Next, steps include implementation evaluation, tool refinement (e.g., a budgeting tool), and a Community of Practice to embed One Health principles, strengthen networks, and prepare graduates for multisector leadership.

Robust training pipelines need enabling structures; the next theme addresses governance and multisectoral coordination.

### Governance and multisectoral coordination

Participants emphasized that implementation at scale in the EMR depends on defined roles, formal coordination arrangements, and aligned policies. Despite years of collaboration efforts, One Health practice still faces major barriers: fragmented surveillance, scarce resources, weak cross-sector coordination, and inconsistent legal frameworks. Uneven institutional capacity and the absence of integrated data platforms further limit action. Donor-driven initiatives have also struggled to endure, which points to the need for context-specific, nationally owned solutions aligned with global strategies such as the One Health Joint Plan of Action (2022–2026) (3, 24–26).

Advancing One Health in the EMR requires tackling linked technical, institutional, economic, and educational issues. Priority technical gaps include limited understanding of integrated health systems, weak disease prioritization, and fractured surveillance and data sharing, which argues for investment in shared frameworks and coordination mechanisms. On the institutional side, advocacy is thin, policies are misaligned, and the roles of One Health professionals are unclear, so governance arrangements and policy alignment must be clarified. Coordination should ultimately produce interoperable surveillance and routine joint risk assessment.

### Surveillance, data integration, and joint risk assessment

The roundtable emphasized interoperable surveillance, joint risk assessment (JRA), and cross-sector rapid response as the operational links between sectors. Minimum integration steps discussed included aligning priority case definitions across human and animal health, adopting a small shared line-list template, and producing a periodic integrated epidemiological summary that combines human, animal, and relevant environmental signals (5, 24, 27, 28). Triggers for action were also highlighted (thresholds to initiate a JRA, criteria for activating cross-sector rapid response teams, and environmental indicators that prompt risk communication). Data governance focused on timeliness, reciprocal access for contributing sectors, and feedback to subnational reporters so that integration leads to decisions and field support. As an illustrative example, management of Rift Valley Fever shows how linking local environmental observations to targeted interventions can strengthen response (10, 29, 30).

As a national illustration, Egypt’s strategy embeds surveillance, JRA, preparedness, and risk communication as core technical pillars supporting early warning and response (31). Specific implementation steps are summarized in Box 1. Sustaining these functions requires predictable resources and aligned budgets; the next theme addresses financing and sustainability.

### Financing and sustainability

Discussions linked sustainability challenges to donor dependency, fragmented funding lines, and limited budget authority for cross-sector activities. In the EMR, economic constraints, particularly in LMICs, highlight the need to embed One Health activities in routine planning and budgeting cycles, assign budget holders for joint tasks (e.g., joint risk assessments, joint exercises, risk communication), and align external support to a single government-led workplan. Educational limitations, such as minimal public awareness, lack of interdisciplinary training, and inadequate curricular integration, further restrict progress (12, 14). Simple process indicators can track whether financing is enabling coordinated action: regular coordination meetings with published minutes, completion of joint risk assessments with action items logged, maintenance of a cross-sector rapid response roster with at least one exercise, and production of a periodic integrated early-warning bulletin (32). With financing aligned, countries are better positioned to act on climate and environmental determinants.

Participants noted that financing remains a persistent bottleneck. In practice, fragmented budget lines across ministries, donor-driven projectization with short time horizons, and procurement rules that limit joint exercises were identified as key barriers. Some participated reported that assigning a designated budget holder for joint risk assessments and pre-authorizing modest cross-sector operating funds have helped sustain coordination.

### Climate and environmental determinants

This theme integrates the climate–health discussion, focusing on heat, air quality, extreme events, and biodiversity-linked risks that should trigger prevention and risk communication across sectors. Climate change, deemed the most significant health threat by WHO (33), exacerbates existing health issues and introduces new risks, including worsening noncommunicable diseases, mental health conditions, altered disease transmission, and disruptions to maternal and child health. Rising healthcare costs and environmental shifts increase pandemic risks, disproportionately affecting vulnerable populations like low-income groups, women, and Indigenous communities (34, 35).

Frequent extreme weather events strain infrastructure and disrupt essential services, reducing health system capacity, particularly in low-resource settings. WHO projects a global shortfall of around 10 million health workers by 2030; this global context underscores the urgency of strengthening One Health workforce pipelines in the EMR (35).

The EMR, with its arid climate and socio-economic disparities, is especially vulnerable (36–38). Although the region contributes only 8.25% of global greenhouse gas emissions, its temperatures rise nearly twice as fast as the global average (39).

Climate shifts exacerbate biodiversity loss, displacement, and health risks, including heat-related illness, air pollution, and waterborne diseases. Vulnerable groups, such as women, children, and refugees, bear the greatest burden (39).

WHO estimates 1 million annual deaths in the EMR are linked to preventable environmental causes, with air pollution accounting for 56% (39). Challenges include limited resources, poor stakeholder coordination, reliance on curative care, and low awareness of climate-health links, compounded by conflicts and emergencies that strain health systems (39).

The “Climate Change, Health and Environment: Regional Framework for Action (2023–2029)” emphasizes building resilient health systems, integrating health into climate policies, improving access to climate funding, and promoting evidence-based policies (39). Its threefold strategy aims to safeguard health, enhance resilience, and reduce emissions while improving outcomes. Collaboration across health ministries, NGOs, and sectors like energy and water is key, with civil society and youth engagement central to driving inclusive solutions (39).

Investments in renewable energy, surveillance systems, and climate-resilient infrastructure are essential. For instance, targeted climate actions in Pakistan could cut emissions by 27.5% by 2030, preventing 65,000 annual deaths from air pollution (39). Through mitigation and adaptation, the EMR can address interconnected health and climate challenges effectively and equitably (39).

BOX 1 **Case study: one health in Egypt: progress, challenges, and way forward**

Egypt began applying the One Health approach in the early 2000s by promoting multisectoral collaboration. A major milestone was hosting the 2008 International Ministerial Conference on Avian Influenza (IMCAPI) in Sharm El Sheikh, which advanced the “One World, One Health” concept. The event stressed global cooperation, fair resource sharing, and sustained political, research, and donor engagement to address HPAI, pandemic threats, and emerging zoonoses (40). By 2011, Egypt became the first country to operationalize the Four-Way Link (4WL), a joint initiative by FAO, OIE, and WHO to enhance national zoonotic disease surveillance and response. The 4WL framework integrates data from epidemiology, laboratory, animal health, and human health systems to support information sharing, joint risk assessment, and coordination. Participants included national epidemiologists, laboratory experts, and representatives from key ministries and international agencies (41, 42).

In 2015, Egypt reinforced its engagement with the Four-Way Link (4WL) approach through Prime Minister’s Decree No. 101, which established a national supreme committee to oversee avian influenza surveillance and coordination. This was followed in 2018 by the creation of the One Health Technical Advisory Group (OH-TAG), tasked with enhancing cross-sector collaboration, facilitating data exchange, improving communication, and addressing antimicrobial resistance while advancing One Health strategies for more effective disease control (41). Most recently, in 2023, the launch of the National Strategy for One Health reinforced Egypt’s dedication to addressing interconnected health threats at the human–animal–environment interface (31).

Egypt’s National Strategy for One Health (2023–2027) consists of a Strategic Framework and an Operational Plan aimed at addressing health risks at the human-animal-environment interface. The framework sets out the overarching vision, mission, and priorities, while the operational plan defines specific actions and policies grounded in One Health principles. The strategy emphasizes collaboration, zoonotic and vector-borne disease control, antimicrobial resistance, food and water safety, and environmental integration. Its technical pillars (such as governance, capacity building, surveillance, joint risk assessment, preparedness, risk communication, cross-border coordination, and research) form the foundation for multisectoral action (31).

The strategy is supported by a hierarchical governance structure led by a Ministerial Committee composed of ministers and institutional heads responsible for policy direction and strategic oversight. Below it, the Supreme Coordination Committee for One Health facilitates cross-sector coordination, guides strategy development, and ensures alignment with national priorities, serving as a link between high-level decision-making and technical implementation (31).

Egypt has undertaken a series of actions to implement its National One Health Strategy, with a focus on strengthening governance, coordination, and technical capacity. A Supreme Coordination Committee and sectoral Technical Working Groups were established, alongside a dedicated One Health department within the Ministry of Health and Population. Capacity-building initiatives targeted zoonotic diseases and climate-related health risks, including the 2024 pilot of the Frontline One Health FETP in partnership with WHO and EMPHNET. Surveillance and preparedness efforts involved assessing national capacities, developing an integrated early warning system, and initiating plans for One Health Rapid Response Teams in collaboration with key ministries. Risk communication and community engagement were advanced through public awareness campaigns and the development of a communication strategy on antimicrobial resistance. Cross-border cooperation was also pursued to address regional health risks. In research, a memorandum with the Ministry of Higher Education formalized collaboration, leading to the identification of 50 national One Health research priorities and the development of a forward-looking agenda to support evidence-informed policy.

Ongoing challenges include securing predictable financing, finalizing data-sharing arrangements for routine joint risk assessment, and sustaining workforce capacity beyond pilot trainings. Immediate next steps echo the roundtable recommendations: formalize regular multisector meetings with published minutes, complete at least two JRAs with a common template, maintain a cross-sector rapid response roster with one joint exercise, and pilot an integrated early-warning brief combining human, animal, and environmental signals.

Despite progress, Egypt’s One Health implementation still faces systemic barriers such as data-sharing gaps, limited coordination frameworks, fragmented budgets, and funding constraints. These illustrate that even with high-level endorsement, sustaining action is difficult. Egypt’s pathway is feasible, but generalizability across the EMR is limited by differences in political commitment, institutional maturity, and resource availability.

### Conclusion

The EMPHNET roundtable on advancing One Health in the EMR highlighted how shared regional challenges can be translated into practical steps. Participants emphasized that effective implementation depends on sustained workforce investment, clear and accountable governance, interoperable surveillance with joint risk assessment, predictable financing, and integration of climate and environmental determinants into health planning. Egypt’s national experience illustrated how One Health can move from concept to structured governance and practice. By embedding One Health in national strategies while fostering regional collaboration, countries in the EMR can strengthen preparedness and build resilient, inclusive, and sustainable health systems.

### Recommendations

Key recommendations emerging from the roundtable include:

1. Establish a regular coordination mechanism with documented agendas and minutes (25, 26).
2. Conduct routine joint risk assessments using a standardized national template and log action items (30).
3. Maintain a cross-sector rapid response roster and conduct at least one joint exercise with after-action review.
4. Develop integrated early-warning products that combine human, animal, and environmental data (29).
5. Approve clear terms of reference for national One Health coordination platforms.
6. Assign or designate budget holders for joint activities to ensure sustainability.
7. Scale national workforce development programs (e.g., FETP-Frontline, FETPV/ISAVET, COHFE-aligned curricula) with mentoring structures.
8. Integrate One Health into national climate policies and preparedness planning.
9. Participate in regular cross-border simulations and regional knowledge-exchange events.

### Limitations

This Perspective is based on a single EMPHNET-convened roundtable and may be subject to convenor bias. Rapporteurs used a structured template, findings were reported in aggregate, and non-EMPHNET co-authors reviewed the synthesis. Attendance was not registered at the room level; the figure of ~68 reflects contemporaneous observation and should be interpreted qualitatively. No triangulation, inter-rater statistics, or participant validation were performed, so findings should be viewed as practice-informed signals rather than definitive estimatses. Some themes received less discussion time and are therefore supported more by secondary literature, though illustrative roundtable examples were retained where possible.
